# Weak smoking cessation awareness in primary health care before surgery: a real-world, retrospective cohort study

**DOI:** 10.1080/02813432.2020.1717093

**Published:** 2020-02-04

**Authors:** Helene Gräsbeck, Heikki Ekroos, Kimmo Halonen, Tuula Vasankari

**Affiliations:** a Department of Pulmonary Medicine, Porvoo Hospital, Porvoo, Finland;; b Doctoral Programme of Clinical Research, University of Helsinki, Helsinki, Finland;; c Department of Surgery, Porvoo Hospital, Porvoo, Finland;; d Department of Pulmonary Diseases and Clinical Allergology, University of Turku, Turku, Finland;; e Finnish Lung Health Association (FILHA), Helsinki, Finland

**Keywords:** Smoking cessation, preoperative period, primary health care, physicians, primary care, surgery

## Abstract

**Objective:** Tobacco smoking is a well-established risk factor for postoperative complications. Research on preoperative smoking cessation in primary health care is scarce.

**Design:** This was a retrospective cohort study.

**Setting:** The *Stop Smoking before Surgery Project* (SSSP) started in Porvoo, Finland, in May of 2016, involving both primary health care and specialized health care. The goals of the project were smoking awareness and preoperative smoking cessation.

**Subjects:** Our study involved 1482 surgical patients operated at Porvoo Hospital between May and December of 2016.

**Main outcome measures:** We studied the recording of smoking status in all patients, and ICD-10 diagnosis of nicotine dependency and the initiation of preoperative smoking cessation in current smokers. Variables were studied from electronic patient records, comparing primary health care referrals and surgical outpatient clinic records.

**Results:** Smoking status was visible in 14.2% of primary health care referrals, and in 18.4% of outpatient clinic records. Corresponding rates for current smokers (*n* = 275) were 0.0 and 8.7% for ICD-10 diagnosis of nicotine dependence, and 2.2 and 15.3% for initiation of preoperative smoking cessation. The differences between primary health care referrals and outpatient clinic records were statistically significant for all three variables (*p* ≤ .001).

**Conclusion:** In primary health care, very little attention was paid to preoperative smoking cessation. Rates were significantly better at the surgical outpatient clinic, but still low. We could not demonstrate any certain effect of the intervention. Our results call for future research on ways to improve smoking cessation rates.

**Key points**
- Tobacco smoking is a well-established risk factor for postoperative complications. Research on preoperative smoking cessation in primary health care is scarce.
- We found weak smoking awareness and weak smoking cessation intervention numbers among both primary and specialized health care doctors. Our results indicate an urgent need for an efficient preoperative smoking cessation model involving both primary and specialized health care.

## Introduction

Smoking is a major cause of morbidity and mortality, and a significant economic burden both in developed and in developing countries. Nicotine, carbon monoxide and several oxygen species of cigarette smoke cause vasoconstriction, reduce oxygen transport capacity of red blood cells, prevent normal wound healing and make tissues susceptible to infections [1].

Large cohort studies on major surgical procedures have demonstrated the increased risk of postoperative complications caused by smoking. They have confirmed higher mortality, and increased risk of cardiac, arterial and pulmonary events and infections among smokers compared to nonsmokers [2–4].

Research has recognized surgery as a ‘teachable moment’ for smoking cessation. The surgical procedure can act as a motivator for healthy lifestyle changes. The probability of quitting smoking is especially high in major surgical procedures, but also noteworthy in outpatient procedures [5]. In elective procedures, the preoperative time frame is usually sufficiently long for smoking cessation in time to provide positive effects regarding postoperative complications. The ideal time frame for quitting is still under debate [6].

The Finnish Current Care Guidelines on Tobacco and nicotine dependence, prevention and treatment recommend that surgical patients should be offered smoking cessation support already when planning for surgery [7].

In most countries, general practitioners (GPs) of primary health care refer patients to secondary care for elective surgical procedures. Ideally, the GP could start the process of smoking cessation already when making the referral. Smoking cessation interventions in primary health care before surgery have been investigated in only one study. The authors concluded that there is an urgent need for development of co-operation strategies between primary and secondary health care to promote preoperative smoking cessation [8].

The goal of our study was to investigate smoking cessation practices among GPs in primary health care and MDs of a surgical outpatient clinic. We focused on patient records’ written information on smoking status, use of ICD-10 code of nicotine dependency F17.2 (ICD-10 ND), and smoking cessation interventions in the baseline situation. We compared primary and specialized health care, since we aimed to demonstrate where the need to improve smoking cessation practices is the most urgent. The other aim of our study was to evaluate the effect of an educational intervention on the target groups.

## Material and methods

The *Stop Smoking before Surgery Project* (SSSP) began in May of 2016 in Porvoo, Uusimaa, Finland. The project was an initiative of the Surgical Clinic at Porvoo Hospital. Porvoo Hospital is a regional hospital caring for a population basis of approximately 100,000 citizens and performing various surgical procedures.

The goals of the SSSP were smoking awareness and preoperative smoking cessation. The recording of ICD-10 ND in patient records of current smokers served as measurement tool for smoking awareness. To achieve these goals, the Chief Surgeon of Porvoo Hospital held lectures at the local primary health care centers of the public sector, and at a staff meeting event of the hospital. The lectures provided information about risks of smoking in the perioperative period, tools for smoking awareness, and benefits of and best practice for preoperative smoking cessation. The lecturer requested physicians to record ICD-10 ND in smokers’ patient records. In the public primary health care units, lectures were held in May and June, and at the hospital in September of 2016. No lectures were held in private sector health care units. The executioners of the SSSP provided written information on hazardous effects of smoking and benefits of smoking cessation in the preoperative period to the public primary health care units and the Porvoo Hospital units. During 2016, regular emails reminded physicians of the public health care sector in the Porvoo area of the SSSP.

This was a real-world, retrospective cohort study approved by HUS (Health Care District of Helsinki and Uusimaa). The material included all patients aged 16 and over undergoing elective and urgent surgical procedures at Porvoo Hospital between May 1st and December 31st, 2016. The first subject studied had undergone surgery on May 2nd and been referred from primary health care on February 2nd. We excluded emergency cases, since they need surgery within hours to a few days, and there practically is no time for preoperative smoking cessation in their case. Numbers of patients of the different surgical specialties are shown in Table 1. Electronic patient records served as the information source on the variables analyzed. The primary and the specialized health care in the Porvoo area do not have joint electronic patient records, and due to this we utilized the specialized health care records and the primary health care referrals. Three clinical research nurses collected the data, which were then evaluated by a trained physician. We studied the recording of smoking status concerning the whole study population, and ICD-10 ND and the initiation of preoperative smoking cessation concerning current smokers. If there was any mention of a cessation intervention (provided information and/or support, nicotine replacement therapy (NRT), smoking cessation medication) we considered it initiation of smoking cessation. We defined patients having smoked cigarettes within six months prior to surgery as current smokers, and patients who had quit smoking at least six months before surgery as ex-smokers. We considered patients with no reported smoking history never-smokers. We obtained each patient´s smoking history data from the whole patient record.

**Table 1.** Distribution of surgical specialties.

Surgical specialty	*n* (%)
Orthopedics	673 (45.4)
Urology	204 (13.8)
General surgery	212 (14.3)
Gastroenterology	188 (12.7)
Plastic surgery	85 (5.7)
Mammary gland surgery	59 (4.0)
Vascular surgery	39 (2.6)
Endocrine surgery	17 (1.1)
Other	5 (0.3)

Variables were analyzed both from the primary health care referrals (PCRs) and the surgical outpatient clinic records (OCRs) of the preoperative visits to compare unit outcomes. In addition, we compared outcomes between primary health care units. These units were the public health care centers in the Porvoo area, and of the private sector, the occupational health care and private practitioners. We also included the hospital´s internal referrals. A total of 152 patients had been referred from other units than the ones previously mentioned, for example directly after breast cancer mammography screening. These subjects were included in the overall comparison between PCRs and OCRs, but we did not compare these other units with the four main referring units in the PCR comparison. Since we were interested in possible intervention effects, we also compared PCRs and OCRs made before April 1st 2016 with those made after October 1st 2016 (before and after the lectures).

IBM SPSS Statistics 25 served as the statistical analysis tool. We studied percent distributions of the variables and compared the PCRs with the OCRs for all three variables, using the paired-samples McNemar test. This test was suitable for the analysis, since the same subjects visited both a primary health care unit and the surgical outpatient clinic. In addition, we compared the referring primary health care units concerning the same variables, using the Chi-square test. *p*-values <.05 were considered statistically significant. When comparing outcomes before and after the lectures, we applied no statistics due to the negative trend.

## Results

A total of 1482 patients were eligible for the study. Of these, 275 (18.6%) classified as current smokers. Of the PCRs, 712 had been made after May 1st 2016, and 401 OCRs had been recorded after September 1st 2016 (the lecture time points).

The recording of smoking status, concerning the whole study population, was visible in 14.2% of the PCRs, and in 18.4% of the OCRs (Table 2). The difference was statistically significant in favor of the outpatient clinic.

**Table 2.** Comparison between primary health care referrals and outpatient clinic records. Smoking status concerns all subjects, ICD-10 ND and smoking cessation current smokers (*n* = 275).

	Smoking status *n* (%)	ICD-10 ND *n* (%)	Smoking cessation *n* (%)
Primary care referrals	211 (14.2)	0 (0.0)	6 (2.2)
Outpatient clinic records	273 (18.4)	24 (8.7)	42 (15.3)
	*p* = .001	*p* < .001	*p* < .001

Concerning current smokers, we did not find ICD-10 ND in a single one PCR. In the OCRs, the number was 8.7%. Preoperative smoking cessation for current smokers was visible in 2.2% of the PCRs, and in 15.3% of the OCRs. The differences between PCRs and OCRs were statistically significant for both variables.

In the comparison of the primary health care units, smoking status recording for all subjects varied between 4.0 and 24.9%, the lowest in occupational health care referrals, and the highest in public health care center referrals (Table 3). The initiation of smoking cessation among current smokers varied between 0.0 and 4.0%.

**Table 3.** Comparison between primary health care units. Smoking status concerns all subjects, ICD-10 ND and smoking cessation current smokers (*n* = 275).

	Smoking status *n* (%)	ICD-10 ND *n* (%)	Smoking cessation *n* (%)	Current smokers *n*
Public health care center	168 (24.9)	0 (0.0)	3 (2.5)	118
Occupational health care	5 (4.0)	0 (0.0)	1 (4.0)	25
Private practitioner	25 (7.0)	0 (0.0)	1 (1.5)	67
Internal referral	7 (4.1)	0 (0.0)	0 (0.0)	36
	*p* < .001		*p* = .686	

When comparing the PCRs made before April 1st 2016 with those made after October 1st 2016, we found no improvement in smoking status (14% and 9%), ICD-10 ND recording (0% and 0%), or smoking cessation initiation (0% and 0%). The same was true for the OCRs (21% and 15%, 4% and 7%, and 14% and 7%, respectively).

## Discussion

Overall, we found very low rates of recording of smoking status and ICD-10 ND, and scarce preoperative smoking cessation interventions. The surgical outpatient clinic performed better than the primary health care units, but still showed overall weak results. Somewhat surprisingly, ICD-10 ND was not visible in one single primary health care referral. It was a main tool of smoking awareness in the SSSP, but this message had apparently failed to reach the target group in primary health care.

Strengths of our study are real-world patient data and a big sample size. In addition, the range of surgical specialties in our study was wide. There are also important limitations of our study. Firstly, a large number of the PCRs and OCRs had been made before the lectures. Secondly, we did not obtain demographic data on the physicians of the primary health care units and the surgical outpatient clinic. This was largely due to the rapid turnover of physicians at these units, a problem that makes it difficult to obtain data valid during the whole study period. Thirdly, we did not interview the physicians concerning attitudes toward smoking cessation and viewpoints on barriers for this. Fourthly, our study relied on self-reported smoking status, a current smoker being defined as a subject having smoked cigarettes within six months before surgery. Due to this, there could possibly have been a fraction of subjects among the current smokers who had actually quit smoking before the planning of surgery. This may have exaggerated the low recording rates of ICD-10 ND and smoking cessation.

In our study, 18.6% of the patients classified as current smokers. This is consistent with other studies on major surgery, where the current smoker proportion has varied between 10 and 42% [9–11]. In the general Finnish population of 20-84-year-olds, the prevalence of daily smoking in 2017 was 13% for men and 10% for women [12]. This prevalence is smaller than both in our study and in the literature. A reason for this may be greater morbidity among smokers, leading to the need for surgical treatment of smoking-related conditions.

To the best of our knowledge, no studies on the recording of preoperative smoking status and ICD-10 ND in primary health care exist. In a study conducted by Warner et al., 90% of the surgeons claimed to always ask the smoking status of their patients before surgery [13]. In our study, smoking status appeared in less than 20% of the OCRs. These two different measurement methods cannot be directly compared, but one can still draw the conclusion that surgeons in our study paid less attention to smoking cessation than those in Warner´s. Reasons for the weak ICD-10 ND registration numbers may be lack of time and difficulty to make it a part of practice routines.

We found that very little attention was paid to preoperative smoking cessation in primary health care. This is consistent with Tonnesen et al.’s exploratory prospective trial, in which GPs were given the opportunity to refer surgical patients to a preoperative program for alcohol and smoking cessation at the surgical clinic. They found an increase in referrals from 0% to only 10% [8].

Initiation of smoking cessation was visible in 15.3% of our OCRs, which is a strikingly low number, considering that previous studies have found 58 to 70% of surgeons to advise their patients to quit smoking before surgery [13–16]. Insufficient training, lack of knowledge, lack of perceived efficacious interventions, and lack of time have proven the most significant smoking cessation barriers for surgeons [13,16,17].

The better outcomes in the OCRs compared to the PCRs may reflect effects of legal aspects, since the responsibility for postoperative complications lies with the surgical clinic. In addition, physicians at the surgical outpatient clinic may have felt more concerned by the SSSP than primary health care physicians did, since the project was an initiative of the surgical clinic. Still, one has to bear in mind that the hospital lecture was held late in the year. Among the primary health care units, public health care centers demonstrated better smoking status recording rates than private-sector units. One may speculate whether this was due to the SSSP, or whether public health care center physicians had more smoking cessation experience than those at private health care units.

Our results demonstrate that very little attention is paid to preoperative smoking cessation in primary health care. The situation of the surgical outpatient clinic appears to be better, but we still found strikingly weak results in an international comparison. When comparing outcomes before and after the lectures, we could not demonstrate any intervention effect. However, when limiting the analyses to these time periods, the subject numbers were small, and the emails and the written information in our intervention could not be noticed and considered properly. The rapid physician turnover may also be a reason for the lack of intervention effect. In conclusion, the baseline smoking cessation habits among MDs seem to be weak, and these preliminary results of the SSSP in its first implementation year demonstrate the need for future interventions to improve smoking cessation numbers.

Preoperative smoking cessation in primary health care is a missed opportunity. Initiation of smoking cessation already at referral would provide the patient with more time to quit smoking before surgery. A recent Dutch study investigating primary health care prevention practices in a cardiometabolic disease context found group practices and smoking cessation programs at the health care centers to be elements improving prevention performance [18]. This could be a feasible approach in the preoperative smoking cessation context as well, taking into account the GP shortage in primary health care. Web-based patient information and support is another solution that has shown promising care results among COPD patients after three months of follow-up [19].

Future research on ways to improve preoperative smoking cessation rates and co-operation between primary and secondary health care on this matter is vital.
